# Role of exosomal ncRNAs in traumatic brain injury

**DOI:** 10.1016/j.ncrna.2023.10.004

**Published:** 2023-10-12

**Authors:** Ozal Beylerli, Rasim Tamrazov, Ilgiz Gareev, Tatiana Ilyasova, Alina Shumadalova, Yunlong Bai, Baofeng Yang

**Affiliations:** a Department of Pharmacology (State-Province Key Laboratories of Biomedicine-Pharmaceutics of China, Key Laboratory of Cardiovascular Research, Ministry of Education), College of Pharmacy, Harbin Medical University, Harbin, 150081, People's Republic of China; b Translational Medicine Research and Cooperation Center of Northern China, Heilongjiang Academy of Medical Sciences, Harbin Medical University, Harbin, 150081, People's Republic of China; c Department of Oncology, Radiology and Radiotherapy, Tyumen State Medical University, 54 Odesskaya Street, 625023, Tyumen, Russia; d Central Research Laboratory, Bashkir State Medical University, Ufa, Republic of Bashkortostan, 3 Lenin Street, 450008, Russia; e Department of Internal Diseases, Bashkir State Medical University, Ufa, Republic of Bashkortostan, 450008, Russia; f Department of General Chemistry, Bashkir State Medical University, Ufa, Republic of Bashkortostan, 3 Lenin Street, 450008, Russia

**Keywords:** Exosomes, Non-coding RNA, Exosomal non-coding RNA, Traumatic brain injury, Biomarkers

## Abstract

Traumatic brain injury (TBI) is a complex neurological disorder that often results in long-term disabilities, cognitive impairments, and emotional disturbances. Despite significant advancements in understanding the pathophysiology of TBI, effective treatments remain limited. In recent years, exosomal non-coding RNAs (ncRNAs) have emerged as potential players in TBI pathogenesis and as novel diagnostic and therapeutic targets. Exosomal ncRNAs are small RNA molecules that are secreted by cells and transported to distant sites, where they can modulate gene expression and cell signaling pathways. They have been shown to play important roles in various aspects of TBI, such as neuroinflammation, blood-brain barrier dysfunction, and neuronal apoptosis. The ability of exosomal ncRNAs to cross the blood-brain barrier and reach the brain parenchyma makes them attractive candidates for non-invasive biomarkers and drug delivery systems. However, significant challenges still need to be addressed before exosomal ncRNAs can be translated into clinical practice, including standardization of isolation and quantification methods, validation of their diagnostic and prognostic value, and optimization of their therapeutic efficacy and safety. This review aims to summarize the current knowledge regarding the role of exosomal ncRNAs in TBI, including their biogenesis, function, and potential applications in diagnosis, prognosis, and treatment. We also discuss the challenges and future perspectives of using exosomal ncRNAs as clinical tools for TBI management.

## 1 Introduction

**Fig. 1:**
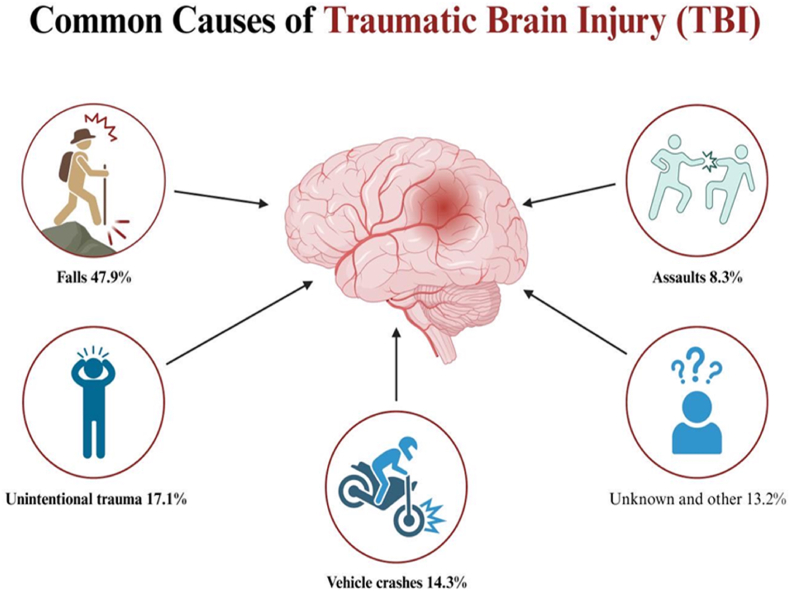
Analysis of epidemiological statistics on the problem of traumatic brain injury (TBI). Patterns in epidemiological changes are observed: currently, falls and unintentional trauma are the most common causes of TBI.

Traumatic brain injury (TBI) is a neurological damage caused by external mechanical force, with over 50 million new cases reported globally each year [1,2]. A large population-based study [3] showed that there are 770,000 to 890,000 new TBI cases in China every year. TBI can result in long-term cognitive impairments and neurodegenerative changes, with no breakthrough in current treatment options [4]. In clinical practice, reducing secondary brain injury, maintaining normal physiological functions, and stabilizing the internal environment are crucial in improving TBI prognosis (Fig. 1) [2].

Extracellular vesicles (EVs) are double-layered phospholipid membrane vesicles with a diameter ranging from 40 to 1000 nm. Based on their size, composition, and biogenesis pathways, EVs can be divided into three types: exosomes, microvesicles, and apoptotic bodies [5,6]. Exosomes are a specific type of EVs with a size range of 50–150 nm. EVs carry various biologically active substances such as nucleic acids, proteins, and lipids and are enriched in exosomes. EVs are secreted by various cells into the extracellular environment and can be detected in various biological fluids [[7], [8], [9]]. The quantity and content of EVs produced by different cells under specific pathological or physiological conditions vary significantly. Moreover, due to the presence of specific adhesion molecules, EVs can deliver their cargo to specific cells, thus regulating the functions of target cells. EVs participate in intracellular communication and material transfer, mediating biological processes such as differentiation, immune response, neural signal transmission, and tumor metastasis [6,10]. Genomic research based on EV contents has great potential in identifying biomarkers, disease treatment, prognosis, and efficacy assessment [11].

**Fig. 2:**
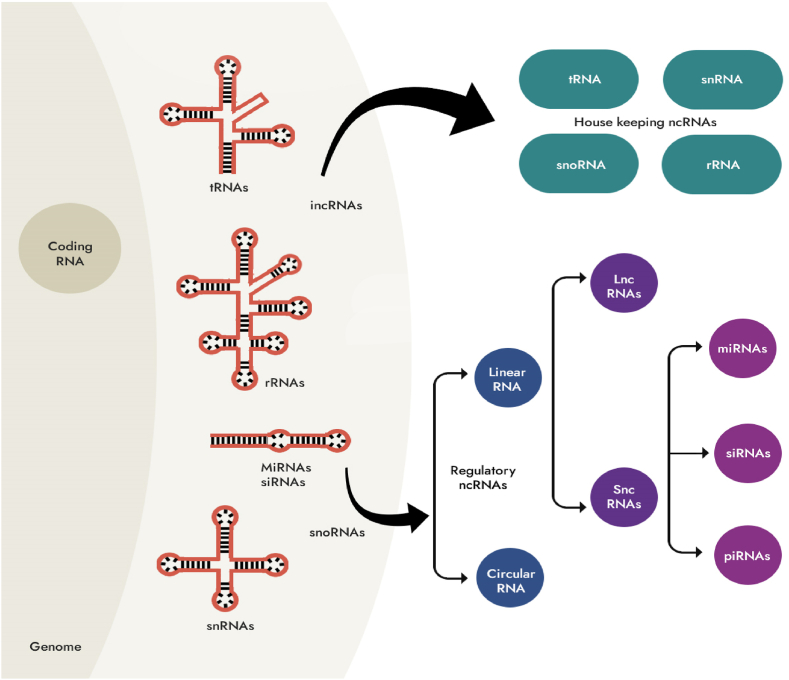
Classification of non-coding RNAs (ncRNAs). Housekeeping non-coding RNAs encompass various types, such as transfer RNAs (tRNAs), small nuclear RNAs (snRNAs), ribosomal RNAs (rRNAs), and small nucleolar RNAs (snoRNAs). On the other hand, regulatory non-coding RNAs comprise microRNAs (miRNAs), short interfering RNAs (siRNAs), piwi-interacting RNAs (piRNAs), and long non-coding RNAs (lncRNAs).

Non-coding RNA (ncRNA) is a type of RNA that does not participate in translation, including microRNA (miRNA), long non-coding RNA (lncRNA), circular RNA (circRNA), etc. The abundant presence of ncRNAs in extracellular vesicles (EVs) has been confirmed to participate in intercellular regulation [12]. After traumatic brain injury (TBI), significant changes in miRNA expression levels have been observed in the cerebral cortex, hippocampus, blood, and cerebrospinal fluid [13,14]; lncRNAs participate in multiple pathological processes after TBI, and are important therapeutic targets and biomarkers (Fig. 2) [15,16]; circRNAs, as regulatory factors, also participate in TBI-related gene expression and cell regeneration processes [[17], [a], [b],^18^]. These findings make extracellular vesicle ncRNAs a promising breakthrough for TBI diagnosis, precision treatment, and prognostic evaluation [16,19,20].

TBI can lead to a series of stress changes in the body, including changes in extracellular vesicles. On the one hand, the number of extracellular vesicles in body fluids increases after TBI, reaching a peak and gradually returning to baseline levels, suggesting that extracellular vesicles are highly involved in various self-regulation processes after injury [21]. On the other hand, the expression of extracellular vesicle contents also undergoes significant changes. The body's response to disease or injury is feedbacked through modifying the contents of extracellular vesicles, especially ncRNAs [22]. For example, after TBI, differentially expressed miRNAs, such as miR-320c, miR-92a, and lncRNA metastasis associated in lung adenocarcinoma transcript 1 (MALAT1), can regulate synaptic activity and neural plasticity [19,23]. In addition, an increase in the expression levels of extracellular vesicle-loaded proteins, such as ubiquitin carboxyl-terminal hydrolase and phospholipid-binding protein-VII, have also been detected, which are involved in the secondary brain injury process [24].

## 2 TBI diagnosis and extracellular ncRNAs

Diagnosis and prognosis of traumatic brain injury (TBI) are complicated by factors such as injury type, location, severity, and individual recovery ability, and conventional diagnostic methods have shown limitations. Extracellular vesicle (EV) proteomics has made significant progress in cancer research, indicating their important role in liquid biopsy [25,26]. EV proteomics analysis also has the potential to differentiate individuals with TBI, but a stable and reliable evaluation standard and system has not yet been established [27]. Due to the resistance of extracellular ncRNA to interference and degradation, they can provide relatively stable and specific biomarkers for disease monitoring [28]. Therefore, correlating these biomarkers with real-time clinical parameters can provide us with clinical information on multiple dimensions of TBI and its recovery, providing opportunities for improving classification, risk stratification, treatment evaluation, prognosis prediction, personalized and even anticipatory treatment strategies [28,29].

Extracellular miRNAs are stable and easy to detect, making them ideal biomarkers for TBI. Harrison et al. isolated extracellular vesicles from the brains of TBI model mice and performed miRNA sequencing, revealing downregulation of miR-212 and upregulation of miR-21, miR-146, miR-7a, and miR-7b, with miR-21 showing the most significant change [30]. Ko et al. developed a method for diagnosing TBI based on extracellular miRNA expression profiles and created a biomarker panel in both rat models and human patients [31]. Results showed that seven miRNAs (miR-129-5p, miR-212-5p, miR-9-5p, miR-152-5p, miR-21, miR-374b-5p, miR-664-3p) could accurately distinguish healthy controls from TBI models with a 99 % accuracy rate. The researchers further conducted an open search for the biomarker panel and successfully classified specific injury states in various injury types, injury intensities, injury histories, injury times, and sham surgery controls in both rat models and clinical samples based on extracellular miRNA profiles [32]. Ko et al. also determined the miRNA biomarkers in extracellular vesicles and their related signaling pathways and found that many pathways were common between preclinical models and clinical samples [32]. Puffer et al. isolated extracellular vesicles from the plasma of TBI patients and identified 11 differentially expressed miRNAs through deep sequencing [33]. The target genes of these miRNAs were highly correlated with pathways related to organismal injury and development, further verifying extracellular miRNAs as TBI biomarkers in humans.

Extracellular circRNAs are also involved in the TBI process and have diagnostic significance. The sequencing spectra of extracellular circRNAs in brain cells after TBI in mice show significant differential expression, with 155 up-regulated and 76 down-regulated [34]. In the cortical expression spectra of circRNAs in a controlled cortical impact (CCI) model of mice, 191 differentially expressed circRNAs were detected. Functional analysis [18] showed that inflammation, cell death, and injury repair were the main biological processes associated with circRNAs.

In the field of neuro-oncology, extracellular lncRNAs are considered ideal diagnostic markers as they are associated with tumor generation, infiltration, metastasis, and chemoresistance, making them useful for early diagnosis [15].

However, there are no reports on the diagnostic potential of extracellular lncRNAs in TBI. It has been demonstrated that traumatic brain injury can induce changes in the expression levels of lncRNAs in vivo. For instance, Zhong et al. found that the expression of 823 lncRNAs in the mouse cortex changed significantly after CCI (667 upregulated, 156 downregulated) [35]. Wang et al. analyzed lncRNA expression in the hippocampus of TBI rats and found that 271 lncRNAs showed differential expression [36]. Functional analysis showed that the most significant changes were in categories related to inflammation, transcription, apoptosis, and necrosis. The associated pathways mainly involved inflammation, cell cycle, and apoptosis. Therefore, the diagnostic potential of extracellularly enriched lncRNAs and circRNAs in TBI warrants further exploration [12].

## 3 Treatment of TBI with extracellular vesicle ncRNAs

The nervous system lacks effective innate healing ability, making neurological repair after injury a major clinical challenge. Extracellular vesicle ncRNA has demonstrated potent repair and regenerative potential in the nervous system [37]. Animal studies have shown the effectiveness of extracellular vesicles in treating neurological damage, as they can promote angiogenesis, reduce inflammation, promote neuroregeneration, and improve neurological function [22].

Mesenchymal stem cell (MSC) transplantation therapy has broad prospects, with its paracrine activity playing a leading role in brain tissue reconstruction [38]. The effect of extracellular vesicles derived from MSCs has been verified [39]. Studies have isolated extracellular vesicles from human bone marrow and used them in rats 24 h after TBI, finding significant inhibition of the activation of GFAP + astrocytes and CD68^+^ microglia/macrophages, thereby exerting an anti-inflammatory effect [39,40]. The therapeutic effect of extracellular vesicles derived from MSCs in the TBI model demonstrates that ncRNA plays a key role in altering the phenotype of recipient cells and regulating biological processes [41]. As a non-cellular therapy, extracellular vesicle therapy based on ncRNA has the advantages of high stability, low immunogenicity, crossing the blood-brain barrier, and targeted transfer to specific cells, making it a new strategy for exploring neural repair after TBI [8,19].

In vitro experiments have shown that extracellular vesicles generated by treating endothelial cells with IL-3 can deliver miR-126-3p and pSTAT5 to recipient endothelial cells, leading to decreased Spred-1 expression, increased ERK1/2 activation, increased cyclin D1 transcription, and ultimately promoting angiogenesis [42]. Han et al. demonstrated that MSC-derived extracellular vesicles significantly improved spatial learning and motor function in rats, with histological observation showing increased numbers of newly formed endothelial cells, differentiated neurons, mature neurons, and myelin sheaths in the hemorrhagic border zone and subventricular zone [43]. Huang et al. revealed that extracellular vesicle-mediated transfer of miR-124-3p from microglia to injured neurons inhibited the activity of the mammalian target of rapamycin (mTOR) signaling pathway, promoting axonal growth, improving hippocampal neurogenesis, and facilitating neurological function recovery [44]. The characteristics of this transfer were an increase in the number and length of dendritic branches, a decrease in the expression of neurodegenerative proteins such as RhoA, amyloid-β-peptide, and p-Tau, and suppression of neuroinflammation, ultimately improving neuronal outcomes.

Secondary TBI also seriously affects the neurological prognosis of patients. In the diagnosis and treatment of TBI, reducing the impact of secondary TBI-related factors, such as neuroinflammatory responses and neuronal apoptosis, is a focus of scientific research and clinical treatment. After TBI, astrocytes and microglia release inflammatory mediators, mediating the process of neuroinflammation [41]. Yang et al. found that bone marrow MSC-derived extracellular vesicles enriched with miR-124 can promote mouse hippocampal neurogenesis, and through inhibiting Toll-like receptor 4 (TLR4) pathway to promote M2 polarization of microglia, thereby inhibiting neuronal inflammation and improving prognosis [45]. Xu et al. found in in vitro and in vivo experiments that extracellular vesicles derived from MSCs successfully inhibited inflammation and promoted neuronal regeneration under the induction of brain-derived neurotrophic factor, and this mechanism may be related to the high expression of miR-216a-5p [46].

Long et al. found that extracellular vesicles enriched with miR-873a-5p can regulate microglial phenotype by inhibiting the NF-κB signaling pathway, alleviate neuroinflammation, and improve post-TBI neurological deficits [47]. Li et al. demonstrated that miR-21-5p in extracellular vesicles can inhibit Rab11a-mediated neuronal autophagy, thereby reducing autophagy-mediated neuronal damage [48]. They used a TBI model and treated HT-22 neurons with brain extracts from the mouse brain to simulate the post-traumatic brain microenvironment and observed HT-22 activation. Results showed that miR-21-5p expression in extracellular vesicles increased in HT-22 and directly inhibited autophagy by targeting the non-coding region of Rab11a. In addition, Li et al. found that extracellular vesicles enriched with miR-124-3p derived from microglia after TBI can transfer to neurons, inhibit neuronal autophagy, and protect neurons [49].

The role of extracellular lncRNAs and circRNAs in TBI treatment has also been confirmed. Patel et al. found that MALAT1 can regulate multiple therapeutic targets, including inflammatory response, and promote neural repair after trauma. Compared to conditions with no extracellular vesicles or those with extracellular vesicles lacking lncRNA MALAT1, human adipose-derived stem cells (hASCs) extracellular vesicles containing MALAT1 significantly improved motor function and alleviated cortical injury in CCI model mice. Moreover, extracellular vesicle lncRNAs from hASCs, such as NEAT1 and MALAT1, played a critical role in promoting endogenous repair, improving motor and cognitive function, and alleviating cortical and hippocampal injury [50]. Extracellular vesicle circRNAs also participated in TBI repair to a certain extent. Studies have shown that extracellular vesicle circRNAs are associated with neuronal growth and repair, as well as the development and signal transduction of the nervous system in mouse brain extracellular environments. It is worth noting that extracellular ncRNAs are also widely involved in the pathological and physiological processes after TBI and have certain value as intervention targets. In summary, using extracellular ncRNAs as potential therapeutic targets for neural injury has clinical application potential and lays the foundation for developing new TBI treatments [50].

Another study proposed to endow artificial materials with the bioactivity of extracellular vesicles. For instance, combining polylactic acid scaffold with extracellular vesicles from human adipose-derived stem cells (hASCs) can enhance bone regeneration and promote the repair of mouse cranial defects [51]. On the one hand, specific molecules loaded in extracellular vesicles can help stem cells migrate to the target injury site; on the other hand, the low immunogenicity of extracellular vesicles can reduce some adverse reactions related to treatment. Although clinical research support is still lacking for this approach, it has great potential for applications in fields such as skull repair and nerve regeneration [52].

## 4 TBI complications and extracellular vesicle ncRNAs

TBI complications are mainly related to post-traumatic bodily stress response and dysfunction of the neuroendocrine system, which can increase patient mortality and affect their prognosis, thus requiring effective control of related complications [53]. Extracellular vesicles are widely involved in the progression of TBI and intercellular communication networks, thereby affecting the occurrence of complications such as epilepsy, osteoporosis, and lung injury [[54], [55], [56]]. Gene regulation related to extracellular vesicle ncRNA may have long-term effects and participate in the occurrence of TBI complications, and related research will provide a theoretical basis for the precise treatment of complications [[55], [56], [57]].

After TBI, extracellular vesicles mediate osteoclast differentiation, leading to bone loss, where extracellular miRNAs are involved in the activation of related pathways. A study isolated extracellular miRNAs from bone marrow and found that compared with the sham surgery group, miRNA-1224 in extracellular vesicles from the TBI group was significantly upregulated, suggesting that extracellular miR-1224 may play a key role in NF-κB activation and osteoclast differentiation after TBI. Targeted inhibition of these signaling pathways may reverse TBI-induced bone loss. However, there are different opinions that extracellular miRNAs play a beneficial role in bone fracture repair during osteogenesis. Additionally, extracellular vesicles can optimize the osteogenic induction of bone marrow MSCs and promote bone regeneration. The author believes that extracellular ncRNAs participate in multiple processes of post-traumatic bone metabolism, and their functions can show diversity. Therefore, specific analysis should be carried out according to different sources and target points of extracellular vesicles during research [51,55,57].

Pulmonary disease is an important factor affecting the prognosis of critically ill patients with neurological conditions, with approximately 30 % of traumatic brain injury (TBI) patients developing acute lung injury (ALI). Extracellular vesicles (EVs) have been shown to participate in the process of ALI following TBI. Serum EV proteins after TBI can activate the neuro-respiratory-inflammatory axis and promote pulmonary microvascular endothelial cell necrosis, thereby inducing ALI [54]. Jiang et al. extracted EVs from the blood of mice with ALI and found that specific miRNAs were enriched, which could induce lung inflammation [58]. This study clarified that EVs deliver miR-155 to macrophages, activate the NF-κB pathway, and induce the production of inflammatory mediators such as tumor necrosis factor-α, interleukin-6, etc. Additionally, EV miR-155 can target SH2-containing inositol phosphatase-1 and cytokine signaling inhibitor-1, promoting macrophage proliferation and inflammatory response.

Post-traumatic epilepsy here is a complication and a risk factor for altered brain recovery. The occurrence of epilepsy involves the control of multiple genes and proteins by miRNA at the systemic level, and miRNA in biological fluids may be a new source of biomarkers for epilepsy [59]. Yan et al. found that 50 miRNAs were differentially expressed in the plasma-derived extracellular vesicles of patients with mesial temporal lobe epilepsy with hippocampal sclerosis compared to healthy controls, and miR-8071 had the highest diagnostic value and could reflect the severity of epilepsy [60]. Karttunen et al. reviewed the role of extracellular vesicles in the diagnosis and treatment of structural epilepsy and suggested that extracellular vesicle miRNAs may be key regulatory factors in epilepsy seizures [56].

## 5 Conclusion

In recent years, studies have revealed new mechanisms of extracellular vesicle ncRNAs in intercellular communication networks [61]. Extracellular vesicle-derived ncRNAs have significant implications for the diagnosis, treatment, and prevention of complications following TBI (Table 1) [[62], [63], [64], [65], [66]].

**Table 1:** Summary of the key benefits and constraints of employing EVs ncRNAs profiling for post-mortem TBI diagnosis.

Advantages	Limitations
**Enhanced Sensitivity:** EVs ncRNA profiling provides heightened sensitivity for detecting molecular changes associated with TBI in post-mortem samples, potentially improving diagnostic accuracy.	**Sample Variability:** The composition of EVs and their ncRNAs cargo can vary among individuals, potentially complicating the establishment of standardized TBI diagnostic criteria.
**Early Detection Potential:** EVs can carry ncRNAs that may indicate TBI at earlier stages, allowing for timely intervention and treatment.	**Tissue Source Specificity:** The origin of EVs can affect their ncRNAs content, necessitating careful consideration of the tissue source when interpreting results.
**Sample Preservation:** EVs and their cargo, including ncRNAs, are relatively stable and less prone to degradation, making them suitable for post-mortem analysis even with degraded tissue samples.	**Temporal Dynamics:** EVs ncRNAs profiling provides static data and may not offer insights into the progression or timeline of TBI, limiting the understanding of the injury's temporal aspects.
**Comprehensive Molecular Insights:** EVs ncRNAs profiling allows for the simultaneous assessment of various types of ncRNAs (e.g., microRNAs, long non-coding RNAs), offering a comprehensive view of molecular alterations in TBI.	**Complex Data Interpretation:** Analyzing EVs ncRNAs data requires advanced computational techniques, and the interpretation of results can be complex, potentially leading to misinterpretations.
**Non-Invasive Options:** EVs can be isolated from bodily fluids like cerebrospinal fluid or blood, providing a minimally invasive approach to post-mortem TBI diagnosis.	**Validation Challenges:** Robust validation of EVs ncRNAs biomarkers is essential to ensure their reliability, and this validation process can be resource-intensive and time-consuming.

**Fig. 3:**
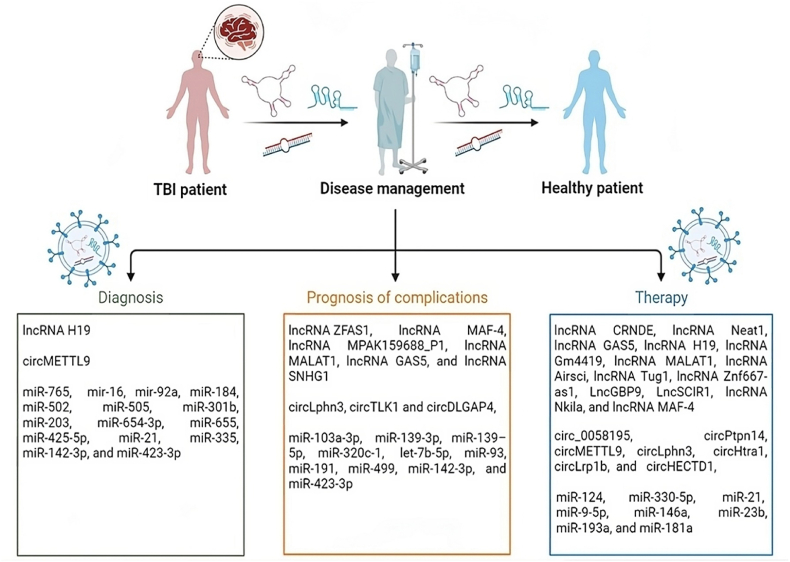
Schematic illustration of the general potential use of exosomal non-coding RNAs (long non-coding RNAs (lncRNAs), circular RNAs (circRNAs) and microRNAs (miRNAs)) in the diagnosis, therapy, and prognosis of potential complications in traumatic brain injury (TBI). Diagnostics confirms the differentiation from other injuries of the central nervous system (CNS). Post-traumatic stress disorder (PTSD) (e.g., disorders of sleep, concentration, memory, and mood), prolonged concussion symptoms (e.g., memory difficulty, headaches, and fatigue), and traumatic optic neuropathy are some of the most common complications of TBI. Therapy involves preclinical studies looking at the network lncRNAs/circRNAs/miRNAs/messenger RNAs (mRNAs).

Future research will focus on the following aspects: 1) conducting omics analysis of extracellular vesicle ncRNAs to develop non-invasive biomarkers for evaluating TBI. 2) Identifying specific groups of extracellular vesicle ncRNAs that promote therapeutic efficacy, studying their modes of action, optimal dosages, treatment time windows, and administration routes, exploring the application of artificial modification of ncRNAs in extracellular vesicle therapy, and conducting personalized extracellular vesicle therapy. 3) Investigating the occurrence mechanism and precise diagnosis and treatment of extracellular vesicle ncRNAs in TBI complications (Fig. 3).

In conclusion, the study of extracellular vesicle ncRNAs in TBI has shown promising avenues. However, before conducting large-scale clinical studies, improvements and refinement of isolation techniques are necessary, as well as a comprehensive understanding of the extracellular vesicle biology features related to the nervous system, to enhance their sensitivity and specificity in the field of TBI application.

## Funding

National Natural Science Foundation of China (NSFC; Grant No. 82170240). This work was supported by the Bashkir State Medical University Strategic Academic Leadership Program (PRIORITY-2030).

## Author contributions

Ozal Beylerli conceptualized and designed the study. All authors have participated in the acquisition, analysis, and interpretation of the data. Rasim Tamrazov and Ilgiz Gareev has drafted the manuscript. Tatiana Ilyasova, Alina Shumadalova contributed to the critical revisions of the manuscript. Yunlong Bai and Baofeng Yang supervised the research. All authors agreed on the journal to which the article would be submitted, gave the final approval for the version to be published, and agreed to be accountable for all aspects of the work.

## Declaration of competing interest

Ozal Beylerli is an editorial board member for Non-coding RNA Research and was not involved in the editorial review or the decision to publish this article. All authors declare that there are no competing interests.

## References

[1] Galgano M., Toshkezi G., Qiu X. (2017). Traumatic brain injury: current treatment strategies and future endeavors. Cell Transplant..

[2] Kaur P., Sharma S. (2018). Recent advances in pathophysiology of traumatic brain injury. Curr. Neuropharmacol..

[3] Jiang J.Y., Gao G.Y., Feng J.F. (2019). Traumatic brain injury in China. Lancet Neurol..

[4] Xiong Y., Mahmood A., Chopp M. (2018). Current understanding of neuroinflammation after traumatic brain injury and cell-based therapeutic opportunities. Chin. J. Traumatol..

[5] Akers J.C., Gonda D., Kim R. (2013). Biogenesis of extracellular vesicles (EV): exosomes, microvesicles, retrovirus-like vesicles, and apoptotic bodies. J. Neuro Oncol..

[6] Margolis L., Sadovsky Y. (2019). The biology of extracellular vesicles: the known unknowns. PLoS Biol..

[7] Antes T.J., Middleton R.C., Luther K.M. (2018). Targeting extracellular vesicles to injured tissue using membrane cloaking and surface display. J. Nanobiotechnol..

[8] Osier N., Motamedi V., Edwards K. (2018). Exosomes in acquired neurological disorders: new Insights into pathophysiology and treatment. Mol. Neurobiol..

[9] Osaki M., Okada F. (2019). Exosomes and their role in cancer progression. Yonago Acta Med..

[10] Kim K.M., Abdelmohsen K., Mustapic M. (2017). RNA in extracellular vesicles. Wiley Interdiscip Rev RNA.

[11] Karnati H.K., Garcia J.H., Tweedie D. (2019). Neuronal enriched extracellular vesicle proteins as biomarkers for traumatic brain injury. J. Neurotrauma.

[12] Yang H., Fu H., Xu W. (2016). Exosomal non-coding RNAs: a promising cancer biomarker. Clin. Chem. Lab. Med..

[13] Yu X., Odenthal M., Fries J.W. (2016). Exosomes as miRNA carriers: formation-function-future. Int. J. Mol. Sci..

[14] Pan Y.B., Sun Z.L., Feng D.F. (2017). The role of microRNA in traumatic brain injury. Neuroscience.

[15] Fan Q., Yang L., Zhang X. (2018). The emerging role of exosome- derived non-coding RNAs in cancer biology. Cancer Lett..

[16] Beylerli O., Gareev I., Sufianov A., Ilyasova T., Guang Y. (2022 Feb 25). Long noncoding RNAs as promising biomarkers in cancer. Noncoding RNA Res.

[17] Sufianov A., Begliarzade S., Beilerli A., Liang Y., Ilyasova T., Beylerli O. (2022 Nov 7). Circular RNAs as biomarkers for lung cancer. Noncoding RNA Res.

[18] Jiang Y.J., Cao S.Q., Gao L.B. (2019). Circular ribonucleic acid expression profile in mouse cortex after traumatic brain injury. J. Neurotrauma.

[19] Atif H., Hicks S.D. (2019). A review of microRNA biomarkers in traumatic brain injury. J. Exp. Neurosci..

[20] Li Y., Zhao J., Yu S. (2019). Extracellular vesicles long RNA sequencing reveals abundant mRNA, circRNA, and lncRNA in human blood as potential biomarkers for cancer diagnosis. Clin. Chem..

[21] Manek R., Moghieb A., Yang Z. (2018). Protein biomarkers and neuroproteomics characterization of microvesicles/exosomes from human cerebrospinal fluid following traumatic brain injury. Mol. Neurobiol..

[22] Sufianov A., Kostin A., Begliarzade S., Kudriashov V., Ilyasova T., Liang Y., Mukhamedzyanov A., Beylerli O. (2023 Feb 7). Exosomal non coding RNAs as a novel target for diabetes mellitus and its complications. Noncoding RNA Res.

[23] Gareev I., Kudriashov V., Sufianov A., Begliarzade S., Ilyasova T., Liang Y., Beylerli O. (2022 Sep 6). The role of long non-coding RNA ANRIL in the development of atherosclerosis. Noncoding RNA Res.

[24] Goetzl E.J., Elahi F.M., Mustapic M. (2019). Altered levels of plasma neuron-derived exosomes and their cargo proteins characterize acute and chronic mild traumatic brain injury. Faseb. J..

[25] Cui S., Cheng Z., Qin W. (2018). Exosomes as a liquid biopsy for lung cancer. Lung Cancer.

[26] Shankar G.M., Balaj L., Stott S.L. (2017). Liquid biopsy for brain tumors. Expert Rev. Mol. Diagn.

[27] Mondello S., Thelin E.P., Shaw G. (2018). Extracellular vesicles: pathogenetic, diagnostic and therapeutic value in traumatic brain injury. Expert Rev. Proteomics.

[28] Taylor D.D., Gercel-Taylor C. (2014). Exosome platform for diagnosis and monitoring of traumatic brain injury. Philos. Trans. R. Soc. Lond. B Biol. Sci..

[29] Goetzl L., Merabova N., Darbinian N. (2018). Diagnostic potential of neural exosome cargo as biomarkers for acute brain injury. Ann Clin Transl Neurol.

[30] Harrison E.B., Hochfelder C.G., Lamberty B.G. (2016). Traumatic brain injury increases levels of miR-21 in extracellular vesicles: implications for neuroinflammation. FEBS Open Biol.

[31] Ko J., Hemphill M., Yang Z. (2018). Diagnosis of traumatic brain injury using miRNA signatures in nanomagnetically isolated brain-derived extracellular vesicles. Lab Chip.

[32] Ko J., Hemphill M., Yang Z. (2020). Multi-dimensional mapping of brain-derived extracellular vesicle microRNA biomarker for traumatic brain injury diagnostics. J. Neurotrauma.

[33] Puffer R.C., Cumba Garcia L.M., Himes B.T. (2020). Plasma extracellular vesicles as a source of biomarkers in traumatic brain injury[J/OL]. J. Neurosurg..

[34] Zhao R.T., Zhou J., Dong X.L. (2018). Circular ribonucleic acid expression alteration in exosomes from the brain extracellular space after traumatic brain injury in mice. J. Neurotrauma.

[35] Zhong J., Jiang L., Cheng C. (2016). Altered expression of long non-coding RNA and mRNA in mouse cortex after traumatic brain injury. Brain Res..

[36] Wang C.F., Zhao C.C., Weng W.J. (2017). Alteration in long non- coding RNA expression after traumatic brain injury in rats. J. Neurotrauma.

[37] Rufino-Ramos D., Albuquerque P.R., Carmona V. (2017). Extracellular vesicles: novel promising delivery systems for therapy of brain diseases. J. Contr. Release.

[38] Li Y., Chopp M. (2009). Marrow stromal cell transplantation in stroke and traumatic brain injury. Neurosci. Lett..

[39] Zhang Y., Chopp M., Meng Y. (2015). Effect of exosomes derived from multipluripotent mesenchymal stromal cells on functional recovery and neurovascular plasticity in rats after traumatic brain injury. J. Neurosurg..

[40] Zhang Y., Chopp M., Zhang Z.G. (2017). Systemic administration of cell-free exosomes generated by human bone marrow derived mesenchymal stem cells cultured under 2D and 3D conditions improves functional recovery in rats after traumatic brain injury. Neurochem. Int..

[41] Yang Y., Ye Y., Su X. (2017). MSCs-derived exosomes and neuroinflammation, neurogenesis and therapy of traumatic brain injury. Front. Cell. Neurosci..

[42] Lombardo G., Dentelli P., Togliatto G. (2016). Activated Stat5 trafficking via endothelial cell-derived extracellular vesicles controls IL-3 pro-angiogenicparacrine action. Sci. Rep..

[43] Han Y., Seyfried D., Meng Y. (2018). Multipotent mesenchymal stromal cell-derived exosomes improve functional recovery after experimental intracerebral hemorrhage in the rat. J. Neurosurg..

[44] Huang S., Ge X., Yu J. (2018). Increased miR-124-3p in microglial exosomes following traumatic brain injury inhibits neuronal inflammation and contributes to neurite outgrowth via their transfer into neurons. Faseb. J..

[45] Yang Y., Ye Y., Kong C. (2019). MiR-124 enriched exosomes promoted the M2 polarization of microglia and enhanced hippocampus neurogenesis after traumatic brain injury by Inhibiting TLR4 pathway. Neurochem. Res..

[46] Xu H., Jia Z., Ma K. (2020). Protective effect of BMSCs-derived exosomes mediated by BDNF on TBI via miR-216a-5p. Med. Sci. Mon. Int. Med. J. Exp. Clin. Res..

[47] Long X., Yao X., Jiang Q. (2020). Astrocyte-derived exosomes enriched with miR-873a-5p inhibit neuroinflammation via microglia phenotype modulation after traumatic brain injury. J. Neuroinflammation.

[48] Li D., Huang S., Zhu J. (2019). Exosomes from miR-21-5p- increased neurons play a role in neuroprotection by suppressing rab11a-mediated neuronal autophagy in vitro after traumatic brain injury. Med. Sci. Mon. Int. Med. J. Exp. Clin. Res..

[49] Li D., Huang S., Yin Z. (2019). Increases in miR-124-3p in microglial exosomes confer neuroprotective effects by targeting FIP200-mediated neuronal autophagy following traumatic brain injury. Neurochem. Res..

[50] Cooper D.R., Borlongan C., Bickford P.C. (2017-11-21).

[51] Li W., Liu Y., Zhang P. (2018). Tissue-engineered bone immobilized with human adipose stem cells-derived exosomes promotes bone regeneration. ACS Appl. Mater. Interfaces.

[52] Yuan J., Botchway B.O.A., Zhang Y. (2020). Combined bioscaffold with stem cells and exosomes can improve traumatic brain injury. Stem Cell Rev Rep.

[53] Lim H.B., Smith M. (2007). Systemic complications after head injury: a clinical review. Anaesthesia.

[54] Kerr N.A., de Rivero Vaccari J.P., Umland O. (2019). Human lung cell pyroptosis following traumatic brain injury. Cells.

[55] Singleton Q., Vaibhav K., Braun M. (2019). Bone marrow derived extracellular vesicles activate osteoclast differentiation in traumatic brain injury induced bone loss. Cells.

[56] Karttunen J., Heiskanen M., Lipponen A. (2019). Extracellular vesicles as diagnostics and therapeutics for structural epilepsies. Int. J. Mol. Sci..

[57] Lu Z., Chen Y., Dunstan C. (2017). Priming adipose stem cells with tumor necrosis factor-alpha preconditioning potentiates their exosome efficacy for bone regeneration. Tissue Eng..

[58] Jiang K., Yang J., Guo S. (2019). Peripheral circulating exosome- mediated delivery of miR-155 as a novel mechanism for acute lung inflammation. Mol. Ther..

[59] Reschke C.R., Henshall D.C. (2015). MicroRNA and epilepsy. Adv. Exp. Med. Biol..

[60] Yan S., Zhang H., Xie W. (2017). Altered microRNA profiles in plasma exosomes from mesial temporal lobe epilepsy with hippocampal sclerosis. Oncotarget.

[61] Sun Z., Yang S., Zhou Q. (2018). Emerging role of exosome- derived long non-coding RNAs in tumor microenvironment. Mol. Cancer.

[62] Sufianov A., Begliarzade S., Ilyasova T., Liang Y., Beylerli O. (2022 Jul 6). MicroRNAs as prognostic markers and therapeutic targets in gliomas. Noncoding RNA Res.

[63] Sufianov A., Begliarzade S., Ilyasova T., Xu X., Beylerli O. (2022 Sep 22). MicroRNAs as potential diagnostic markers of glial brain tumors. Noncoding RNA Res.

[64] Beilerli A., Begliarzade S., Sufianov A., Ilyasova T., Liang Y., Beylerli O. (2022 Jul 31). Circulating ciRS-7 as a potential non-invasive biomarker for epithelial ovarian cancer: an investigative study. Noncoding RNA Res.

[65] Sufianov A., Begliarzade S., Kudriashov V., Beilerli A., Ilyasova T., Liang Y., Beylerli O. (2022 Nov 22). The role of circular RNAs in the pathophysiology of oral squamous cell carcinoma. Noncoding RNA Res.

[66] Begliarzade S., Beilerli A., Sufianov A., Tamrazov R., Kudriashov V., Ilyasova T., Liang Y., Beylerli O. (2023 Feb 21). Long non-coding RNAs as promising biomarkers and therapeutic targets in cervical cancer. Noncoding RNA Res.

